# Assessment of executive functions in school-aged children: A narrative review

**DOI:** 10.3389/fpsyg.2022.991699

**Published:** 2022-11-01

**Authors:** Sofiane Souissi, Karim Chamari, Tarek Bellaj

**Affiliations:** ^1^Aspetar, Orthopaedic and Sports Medicine Hospital, FIFA Medical Centre of Excellence, Doha, Qatar; ^2^Psychology Laboratory, Faculty of Humanities and Social Sciences of Tunis, Tunis University, Tunis, Tunisia; ^3^Department of Biological Sciences, ISSEP Ksar-Said, La Manouba University, Tunis, Tunisia; ^4^Psychology Program, Department of Social Sciences, College of Arts and Science, Qatar University, Doha, Qatar

**Keywords:** evaluation, executive control, cognitive development, pediatric, real-world functioning

## Abstract

**Introduction:**

In the past three decades, there has been increasing interest in assessing children’s Executive Functions (EF). However, studies on the conceptualization and operationalization of this construct are incongruent and guidance for clinicians and researchers aiming to assess EF is insufficient due to measurement variability.

**Aims:**

The purpose of this article was to examine current theories and models of EF in children, identify their assessment instruments, issues, and challenges, and discuss their impact on children’s cognitive, behavioral, social and/or emotional development.

**Methods:**

This narrative review reflected on English and French scholarly articles on EF assessment in children. References were identified through searches of PubMed, Medline, ScienceDirect, Google Scholar, and APA PsychNet throughout the last two decades up to June 2022.

**Results:**

There are commonalities despite divergence in the definition and operationalization of EF. Assessment of EF requires psychometric tests as well as rating scales that must be integrated and interpreted considering the child’s biological makeup, environmental background, and cultural specificities.

**Conclusion:**

Current EF theories, assessment tools, issues, and challenges were discussed in addition to the impact of their components’ dysfunctions on children’s development. Further studies should be conducted to develop new measurement methods and technologies to improve the ecological and ethological validity of youth assessment, treatment, and interventions.

## Introduction

Executive Functions (EF) are a commonly cited construct in cognitive, educational developmental and neuropsychology fields. A search of that term in PubMed, over the last two decades by the end of June 2022 yielded 31,296 papers. However, EF remain a controversial topic in research due to the wide range of definitions, theories, and measures associated with it (Baggetta and Alexander, 2016; Soto et al., 2020; Laureys et al., 2022). Studies on the conceptualization and operationalization of this construct are incongruent and guidance for clinicians and researchers aiming to assess EF is insufficient due to measurement variability (Baggetta and Alexander, 2016; Wallisch et al., 2018). Children’s EF remain up for debate, especially in terms of their number, nature, degree of separation, developmental trajectories, and milestones along with the ecological and construct validity of their measures. The objectives of this review were to examine current theories and models of executive functioning in children, identify their assessment instruments, issues, and challenges, and discuss how they impact cognitive, behavioral, and/or social-emotional development in children.

## Materials and methods

Scholarly articles in English and French were searched using PubMed, Medline, ScienceDirect, Google Scholar, and APA PsychNet. Our search terms were “executive functions” AND “assessment” AND “school-aged children” with filters, 2002-2022, age 6-12. The titles, abstracts, and full texts of relevant articles were reviewed for inclusion and their eligibility was determined by two reviewers.

## Results

The databases’ searches yielded 421 articles. Eligibility and availability were determined by two review authors KC and TB. 85 articles published between 2002 and 2022 were considered eligible and were reviewed. Further sources were derived from lists of similar articles and article reference lists. The literature review was supplemented by manuals of the cited measurement tools and a few book chapters relevant to the topic.

## Discussion

### The executive functions construct

Different definitions of EF exist and there is a lack of consensus as to the exact components of this complex construct. Most researchers agree that EF refers to a variety of top-down mental processes including inhibition, working memory (WM), attention, planning, self-monitoring, self-regulation, and initiation (Goldstein and Naglieri, 2014). Therefore, EF are composed of both cognitive and emotional components, and they play a crucial role in the regulation of goal-oriented behavior (Fernández et al., 2014). In adults, converging research supports the unity-but-diversity view and argues that EF have three distinct yet related “core” dimensions: inhibitory control WM and cognitive flexibility (Garon et al., 2008; Friedman and Miyake, 2017). Higher-order EF are built from these, such as planning, reasoning and problem-solving (Diamond, 2013). In children, it has been demonstrated that EF are relatively undifferentiated in preschoolers until about the age of 5 (Lee et al., 2013). Indeed, between the ages of 3 and 5, a unitary factor model is commonly observed in early childhood (Brydges et al., 2012; Willoughby et al., 2016). However, after age 6, inhibition and WM appear to be two distinguishing factors and EF begin to specialize toward a multifactorial structure, as it does in adults (Huizinga et al., 2006; Van der Ven et al., 2013).

According to others, EF have both “cool” and “hot” components. In cool cognitive skills, logic and critical thinking are required under relatively abstract, decontextualized, and non-emotional conditions (Chan et al., 2008; Nyongesa et al., 2019). For example, verbal reasoning, planning, problem-solving, sequencing, attentional control, inhibition, and behavioral monitoring. Conversely, hot cognitive skills are necessary in contexts that require personal interpretation and involve motivation, emotions or tension between instant gratification and long-term rewards (Zelazo and Müller, 2010). Social cognition, affective decision-making, emotion regulation and the ability to delay gratification are some of the affective cognitive abilities considered an important component of hot EF (Bechara et al., 1999). However, according to Damasio’s somatic marker hypothesis Damasio (1996), emotion and cognition are closely related in EF rather than separate, therefore the hot/cool EF dichotomy cannot be so definitively separated. Indeed, Damasio et al. (1990) argue that without the emotional element it is not possible to engage EF optimally in the real world. The later author speculated that decision-making difficulties observed in the case study of EVR, following Ventromedial Prefrontal Cortex (VPC) damage were caused by his emotional changes in day-to-day living.

The distinction between domain-general and domain-specific EF has also been a source of debate. Baggetta and Alexander (2016) argue that EF could be domain-general or/and domain-specific. They can operate in any domain (e.g., academic performance) or they can affect areas or disciplines more dramatically than others (mathematics vs. reading and writing, for instance). According to Baggetta and Alexander (2016), EF can even simultaneously operate on a general and domain-specific level. It is paramount to note that unitary versus multi-dimensional and/or hot versus cool EF should not be viewed as synonyms for domain-general and domain-specific. It is indeed important to view these analysis levels as distinct but not mutually exclusive.

### Models of executive functions

Executive functions have been conceptualized in a variety of ways, depending on the theoretical point of view and the age range or population studied. EF have traditionally been posited in two ways: the unitary perspective, which stresses common processes among measures of EF, and the modular or componential view, which emphasizes dissociable EF processes and distinct developmental patterns for each process (Toplak et al., 2017). A classic cognitive psychology debate between Marr’s modularity premise Marr (1982) and Fodor’s unitary versus equipotential system Fodor (1983) echoes the distinction between unitary versus modular EF.

Other researchers such as Brock et al. (2009) and Chan et al. (2008) have proposed a classification of EF based on functional criteria by distinguishing between Hot (socio-affective) and cool (cognitive) functions.

The purpose of this article is not to present a comprehensive review of EF’s models. Rather, it examines the most cited and widely followed models based on Baggetta and Alexander (2016) systematic review.

### Unitary view of executive functions

There is substantial evidence to support the unitary view, including consistently high intercorrelations among various measures of EF, especially at younger ages (Friedman and Miyake, 2004; Garon et al., 2008) with a considerable development of the EF between 3 and 6 years of age (Carlson et al., 2004; Friedman and Miyake, 2004). In addition, some factor analytic studies of EF in preschool-aged children showed that EF are largely defined as a single cognitive ability (Hughes et al., 2010).

### Garon’s integrative framework of executive functions

**FIGURE 1:**
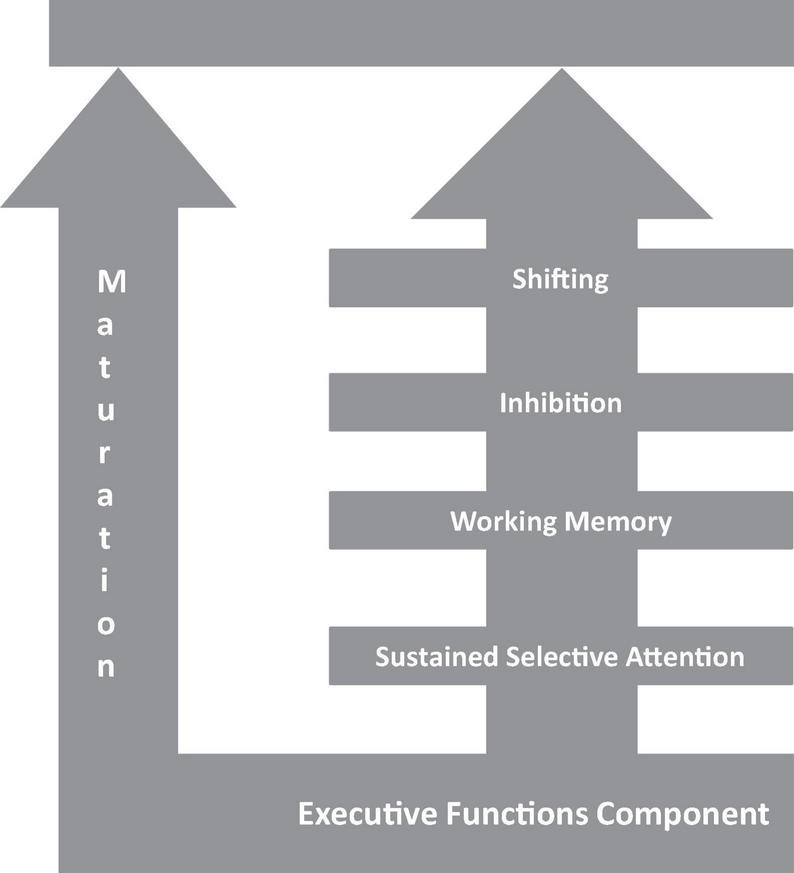
The Developmental Integrative Framework of EF (Garon et al., 2008). Adapted from “Applying an integrative framework of executive function to preschoolers with specific language impairment”, by Kapa et al., 2017, Journal of Speech, Language, and Hearing Research, 60(8), p. 2171.

The developmental Integrative Framework of EF (Garon et al., 2008) is based on a systematic review of the literature on EF in young children using the model developed by Miyake et al. (2000) for EF in adults. The model considers EF to be unitary with partially dissociable components including WM, shifting, and response inhibition in addition to an underlying attention component. Furthermore, the authors describe a hierarchy between EF in which earlier-developed skills, such as attention, support more complex skills, such as shifting which involves the coordination of several EF at once (see Figure 1). According to Garon et al. (2008), attention is the core of all EF abilities. Inhibition comes next in the hierarchy after WM. As children mature, they gain the ability to coordinate various EF components from infancy through early preschool. Children can acquire more complicated EF abilities such as shifting, attention, planning, and problem-solving by coordinating lower-level skills. Although Garon and colleagues’ model takes into account typically developing children, there are significant implications for children with developmental disorders. The hierarchical relationship between EF components suggests that a child who has difficulty with lower-level components would have difficulty with higher-level components as well due to the potential cascade effects.

### Modular view of executive functions

Based mostly on studies of adults and individuals with brain injuries, the modular view postulates that EF can be separated into various relatively independent, functional modules. Thus, neuropsychological batteries frequently include many tests presumably measuring different aspects of executive abilities (e.g., Tower of Hanoi for planning; Wisconsin Card Sort for cognitive flexibility). Although the unitary or common process approach of EF as a model for young children has a lot of support (Garon et al., 2008; Wiebe et al., 2011; Brydges et al., 2012), there is evidence that some components are dissociable. Inhibition, WM and cognitive flexibility, for example, have been shown to develop in diverse trajectories (Diamond, 2006; Best et al., 2009; Bellaj et al., 2016).

### Diamond’s model of executive functions

**FIGURE 2:**
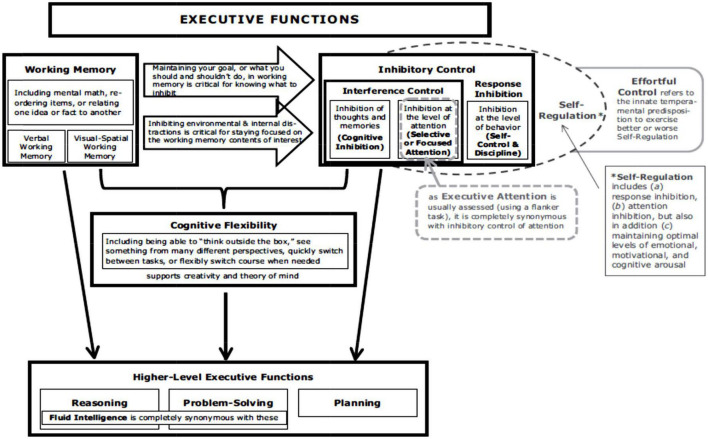
Structure of Executive Processes Based on Diamond’s Proposal Diamond (2013). Reproduced with permission from the Annual Review of Psychology, Volume 64 © 2013 by Annual Reviews, http://www.annualreviews.org.

Another recent theoretical model is illustrated in Figure 2 proposed by Diamond (2013). It is a hierarchical integrative model that illustrates the developmental dynamics and interrelationships between several EF: WM, inhibition, mental flexibility, and higher-level functions such as planning, prioritizing (Romero-López et al., 2017), problem-solving, decision-making (Favieri et al., 2022) and reasoning skills. According to this model, inhibition and WM would emerge early, from the preschool period, while mental flexibility would differentiate from the previous processes later in childhood or even during adolescence. Planning, itself dependent on the former processes, would be differentiated even later. Thus, the cornerstone of EF development would be the processes of inhibition and WM, which would develop relatively early in childhood and would be interdependent.

### Dennis’s model of prefrontal development

Among the theoretical models of EF in children, Dennis (2006) proposes a model of the development of the prefrontal cortex to EF. The Prefrontal Cortex (PFC) will allow, in a top-down process, the formation of linked representations, allowing the elaboration of an action plan. The processing resources that make the establishment of these representations possible will be the WM and inhibitory control, dependent on dorsolateral frontal regions. The content of these representations would refer to knowledge structures that ensure a temporal link, a thought link, and an emotional link. Thus, based on these representations, Dennis (2006) maps the development of the PFC and allocates several functions to it: First, planning, autobiographical memory, and prospective memory attached to the temporal link. Second, metacognition, theory of mind, and social problem-solving are related to the thought link. Finally, affective decision-making, emotional regulation, and disclosure of emotions, are related to emotionallink.

Among the theoretical models of EF in chilDennis’s adolescent model, therefore, places inhibitory control and WM at the core of executive development. These processes are considered to provide various types of links (temporal, thought, emotional). However, Diamond (2013) model is more focused on the four executive processes classically identified in children, structured through a hierarchical and age-differentiated development and interrelationships between the executive components.

In summary, despite the different points of view that the above models claim, most of them agree that EF are multidimensional encompassing different inter-related and interdependent components or functions. Researchers disagreed on what a specific model of EF should be, how many components or processes are involved, and how closely inter-related they are. Researchers have also found several developmental differences in EF (e.g., Wiebe et al., 2011; Brydges et al., 2012) suggesting EF are more unidimensional in early development but becomes a multifactorial construct in adolescence and adulthood and asserting that a single factor model of EF is not universally appropriate across development. Nevertheless, it should be noted that the different findings from some studies are likely due to a wide range of methodologies as well as sensitivity issues in tasks and scoring methods (Van der Ven et al., 2013). These disparities have led to many individual different components. However, in general, a consensus appears to exist regarding three processes: inhibitory control, WM and cognitive flexibility or shifting, planning is sometimes listed as a higher-order EF.

### Development of executive functions

The development of EF depends on the PFC maturation and its networks, including neural fibers myelinization. EF show prolonged development concerning the extensive maturation of the cortical networks that underlie them. EF development, although protracted, emerges rapidly, as early as the first months of life (Diamond and Goldman-Rakic, 1989; Diamond, 2013, 2020). Neuroimaging has revealed structural and functional changes in brain regions underpinning EF during middle age and older adulthood, that are highly likely to affect performance in these age groups (Petrican et al., 2017). A recent study by (Ferguson et al., 2021) examined EF in a large, community-based sample aged from 10 to 86 years old, demonstrating that planning ability and WM capacity continue to develop throughout adolescence as well as in early adulthood.

### Working memory

Early in life, infants and young children are able to hold information in their minds for quite a long time (Diamond, 1995; Nelson et al., 2012). In tasks such as A-not-B, infants of 9 to 12 months can update their WM contents (Diamond, 1985; Bell and Cuevas, 2016). However, memorization and mental manipulation (e.g., reordering visual representations of objects by size) are much more delayed and take longer to develop (Davidson et al., 2006; Cowan et al., 2011). WM in children improves with age due to enhanced interference inhibition (Hale et al., 1997). Children’s performance improves throughout childhood and full capacity of WM may be reached by late adolescence (Gathercole et al., 2004). With aging, WM declines primarily due to a decrease in inhibitory control, making older adults more vulnerable to proactive and retroactive interferences (Solesio-Jofre et al., 2012) as well as distractions (Rutman et al., 2010).

### Inhibitory control

A fundamental aspect of EF is inhibition, which does not seem to be uniform (Best et al., 2009). Hasher and Zacks (1988) posited that the inhibitory processes underlying selective attention are less efficient under certain circumstances. With a decrease in inhibition, irrelevant information can enter WM more easily, and once it enters, it is able to sustain its activation. As a result, memory access rates are reduced. According to Zacks and Hasher (1997), there are three inhibitory functions: the “access function”, which limits access to the WM to those with direct relevance to the task, the “deletion function” removes information from the WM that is irrelevant, inappropriate, or becomes irrelevant due to changes in goals or demands. Finally, the “restrain function” stops the emission of strong responses.

The ability to inhibit most often means suppressing a dominant or automatic response but inhibition can also include interference control, motor control targeted forgetting and emotional control (Nigg, 2000). Furthermore, inhibitory demands seem to vary depending on whether WM is also required, on the response, as well as on the degree of prepotency of the response (Best et al., 2009). Improvements in inhibition are particularly evident in preschool, but also increase between ages 5 and 8 and continue into early adulthood (Romine and Reynolds, 2005). According to Best et al. (2009), in contrast to preschool, adolescence consists mainly of improving speed and accuracy rather than fundamental changes (e.g., learning to consistently inhibit prepotent responses). Inhibitory control abilities may be assessed by using tasks including interference suppression and response inhibition (Young et al., 2017).

### Cognitive flexibility

Cognitive flexibility includes being able to see something from different spatially or interpersonally perspectives, quickly switch between tasks or set-shifting courses when needed (Diamond, 2013). Cognitive flexibility is a core EF that requires and builds on inhibitory control and WM (Davidson et al., 2006; Garon et al., 2008). Simple set-shifting tasks have been observed to be passed with few errors by children as young as 1.5 years old (Stahl and Pry, 2005). According to Jordan and Morton (2012), it is not until children begin to demonstrate cognitive flexibility in the late preschool years that they are capable of executing more challenging tasks (e.g., tasks with complex rules). After kindergarten, there are even more improvements (Luciana and Nelson, 1998), which last until early adolescence, the time when they reach adult levels.

### Planning, reasoning, and problem solving

According to Diamond (2013), a high-level EF are a synonym of fluid intelligence. This skill includes the ability to plan and solve problems as well as inductive and deductive logic reasoning. Planning is an essential part of goal-directed behavior; it involves formulating actions in advance and approaching tasks in a strategic, organized, and effective manner (Anderson, 2002). As children face new situations, planning directs and evaluates their behavior. Das et al. (1994) have shown that children are required to plan several steps of actions in advance, evaluate those actions, and change course, if necessary, when completing planning tasks. Among the most widely used tests are those for the Tower of Hanoi (TOH) and the Tower of London (TOL). Performance in planning seems to improve over time, at least into late childhood and often into adolescence (Best et al., 2009).

### Factors impacting the child executive functions development

Multiple factors can affect one’s ability to complete EF-related tasks successfully. Friedman et al. (2008) state that genetics may explain a significant part of certain EF skills, including inhibition and shifting. Genes linked to EF include catechol-methyltransferase (COMT) and reelin genes (Baune et al., 2010; Logue and Gould, 2014). Environmental factors influencing EF include maternal depression during pregnancy and exposure to teratogens, such as drugs, alcohol, lead, mercury, and pesticides (Hughes et al., 2013). Additionally, harsh rearing, neglectful parenting, and/or low cognitive stimulation during childhood may contribute to poor EF skills throughout the lifespan (Rhoades et al., 2011). Cultural factors also affect EF ability. Indeed, the cultural characteristics (e.g., breadth of the environment, language & writing, languages spoken, level of education) play a major role in psychological, social, and even neural development (Bellaj, 2011; Bellaj et al., 2016; Guerra et al., 2020). It has been demonstrated that socioeconomic status consistently predicts EF by altering neural development through stress, infection, poor nutrition, and/or other factors (Noble et al., 2007). The above environmental factors likely influence EF by turning on or off genes that underlie EF skills through epigenetic changes (Champagne, 2010). In other words, the environmental influences - positive and/or negative children’s experiences - can affect the expression of their genes.

### Executive dysfunction in school-aged children

Attention deficit hyperactivity disorder, traumatic brain injury, autism spectrum disorder, learning disabilities and conduct disorder are associated with deficits in EF (Gioia et al., 2002). Each of these disorders has a distinct profile of EF deficits (Pennington and Ozonoff, 1996). However, these deficits can vary and increase in severity with eventual co-occurring diagnoses (Benallie et al., 2021).

Executive functions problems can be expressed in several ways. It is common for children to act impulsively and have trouble stopping activities, to struggle getting started and remaining engaged, or to become easily distracted and not stay focused. EF impairments are often global, impacting every aspect of behavior (Lezak et al., 2012). The difficulty of EF can be detrimental to academic performance in novel situations or when complex problem solving is necessary (Best et al., 2011).

“Attention deficit hyperactivity disorder (ADHD) is a neurodevelopmental disorder characterized by symptoms of inattention, hyperactivity, and/or impulsivity” (American Psychiatric Association [APA], 2013). ADHD has been described as an EF disorder given the key role executive dysfunction plays in the hyperactive and inattentive behaviors found in children with ADHD (Barkley, 1997). Many researchers attributed the deficits observed in children with ADHD, including WM, affect regulation, motivation, and problem solving to inhibition deficiency. Barkley (1997) argued that inhibition is responsible for all the deficits. Similarly, Pennington and Ozonoff (1996) claimed that inhibition and WM are disturbed in ADHD. Also, according to Bayliss and Roodenrys (2000), ADHD symptomatology is due to a supervisory attentional system lacking inhibitory control.

ADHD children with difficulties in EF may have poorer developmental outcomes (Halperin et al., 2008) and especially for poor outcomes in academic functioning (Bierman et al., 2008). For example, Biederman et al. (2006) have found that children with ADHD and coexisting EF deficits show higher levels of inattention and school problems and score lower on intelligence scales than children with ADHD without EF deficits. According to Molfese et al. (2010), WM and inhibitory control deficits seem to contribute to the classroom behavior problems frequently seen in children with ADHD. Rutledge et al. (2012) have assumed that the poor achievement in reading and math, high use of special education services, and low high-school graduation rates observed in children with ADHD could be partially explained by EF deficits.

An alternative view of ADHD is to propose that instead of executive disorder, the behaviors characteristic of ADHD may reflect deviance in motivational style (Hale et al., 1997). It is possible that children with ADHD have altered perceptions of time, which may explain why they are motivated to avoid or escape delay (Sonuga-Barke et al., 1994). In support of this “dual path” model, several experiments (Sonuga-Barke et al., 1996; Solanto et al., 2001) have shown that preferring delayed rewards and inhibiting control are dissociable. In that regard, behavioral EF can exacerbate school issues by reducing the students’ motivation to succeed (Poletti, 2009).

“Autism spectrum disorder (ASD) is a neurodevelopmental disorder in which the essential features are (i) persistent impairment in reciprocal social communication and social interaction and (ii) restricted, repetitive patterns of behavior, interests, or activities” (American Psychiatric Association [APA], 2013). Autism is associated with high-level (i.e., cognitive) and non-spatial EF impairments (Hughes and Graham, 2002); relatives of autism individuals also display a similar profile of impairments (Hughes et al., 1997). Autistic people tend to exhibit rigid thought patterns associated with poor flexibility (Ozonoff and Cathcart, 1998). Moreover, planning and organizational deficits are common among patients with autism, especially those with high-functioning autism, which is characterized by verbal disorganization (Benallie et al., 2021). According to Minshew et al. (1997), these deficits are due to “weak central coherence” and poor informational processing. In autism, WM impairments are more controversial (Hughes and Graham, 2002). There is considerable variability in autistic impairments in WM and while verbal WM skills may be weak, spatial WM skills may be average or above average. Deficiencies in metacognition and initiation may partially explain poor adaptive functioning commonly seen in this population (Gilotty et al., 2002).

Patients with Traumatic Brain Injury (TBI) also exhibit deficits in EF. Damage severity and location will determine the severity of impairments (Gioia et al., 2002). Generally, disruptions in EF as a result of TBI can be attributed to changes in frontal lobe systems, including damage to white matter tracts. Damage to the dorsolateral prefrontal cortex, orbitofrontal prefrontal cortex, and anterior temporal lobes may also place individuals at great risk of executive dysfunction (Levin et al., 1993). Early-life brain injuries can cause more severe EF disruptions by preventing mastery of EF skills (Riccio et al., 2010) and executive dysfunction often persists many years after the injury (Dawson and Guare, 2018). Deficits in attention, memory, processing speed, flexibility, inhibition, planning, organization, and self-regulation have been observed in individuals with TBI (Dennis et al., 2001).

Learning disabilities, especially reading disorders, may be related to underlying EF deficits (Gioia et al., 2002). WM deficits have been correlated with word recognition and reading comprehension (Swanson and Ashbaker, 2000). Individuals with reading comprehension difficulties are more likely to have additional planning and organizing problems (Levin et al., 1993). Problem-solving, logical reasoning and WM deficits were noted in Children with learning disabilities in math (Riccio et al., 2010). The latter researchers assume that the difficulties to filter out distracting information and select and switch between strategies contributes to problems in mathematic skills. Berk and Diaz (1992) argued the role of EF in other learning disabilities by the fact that the classroom environment presents novel activities and assignments each day, requiring attentional load devoted to problem solving. The hypothesis of motivation disruption was also put forward by Barkley (2012), resulting in difficulty initiating school-related activities and projects. In other words, children with EF deficits can show symptoms in the classroom that prevent them from succeeding such as poor motivation, self-starting behavior, attention to assignments, and lack of problem-solving skills.

Thus, originally described in frontal lesions, dysexecutive syndromes now reside in a diffuse network encompassing the prefrontal cortex, parietal cortex, basal ganglia, thalamus, and cerebellum as well. Executive dysfunction can result from injury to any of these areas, their white matter connections, or their neurotransmitter systems. Therefore, dysexecutive symptoms occur in almost all neurodegenerative diseases, and they are also common in many neurologic, psychiatric, and systemic illnesses. An optimal management approach should address the underlying cause as well as maximize patient function and safety (Rabinovici et al., 2015).

### Executive functions assessment in school-aged children

Executive functions has received particular attention in recent years due to its importance in influencing children’s academic, social, and behavioral outcomes. It plays an important role in any comprehensive assessment of children and adolescents (Nyongesa et al., 2019). Furthermore, such assessment can play a critical role in diagnosing certain childhood disorders and in developing interventions to potentially help children with executive dysfunction. According to McCoy (2019), clinical and educational professionals are increasingly recognizing that reliable and valid evaluation measures of EF are necessary for children, which led to the use of performance-based tests and rating scales (parents, teachers, and clinicians). In the following sections, we will review the most cited and commonly used EF assessment tools for children followed by the advantages and disadvantages of each of these psychometric assessment methods, and finally some of the challenges to executive functioning assessment.

### Performance-based tests

These tests are called neuropsychological, psychometric, or performance-based tests and are typically used in clinical and research contexts. The measures involve individual tasks or battery of tests assessing various aspects of the subject’s performance. The response times, as well as the number of errors and omissions, are among the measured indices (Fernández et al., 2014). Some of the widely used individual tests with school-aged children measuring WM include the Digit Span Tests (forward and in reverse order) from the WISC-V subtests (Wechsler, 2014). The Corsi’s block-tapping test which is a classical non-verbal test to assess visuospatial WM (Corsi, 1972), the Dot Matrix within the Automated WM Assessment battery (Alloway, 2007), and finally the n-back pictorial paradigm gradually increases the WM load from 0- to 1- to 2-back (Isquith et al., 2014).

Several variations of the Stroop Test (Stroop, 1992; Fernández et al., 2014), including the Five Digit Test: FDT (Lang, 2002), the Day-Night Stroop test (Gerstadt et al., 1994) and the Animal Stroop test (Wright et al., 2003) have been extensively used for measuring inhibition in school-aged children. Another tool considered as a “gold standard” measure of response inhibition is the go/no-go task (Georgiou and Essau, 2011). In this task, the “Go” stimuli require an automatic or prepotent response from the user, such as a key press on the keyboard. Conversely, responding to a no-go stimulus must be inhibited. Among the tests including the go/no-go paradigm are the Continuous Performance Test (CPT), the Test of Variables of Attention (TOVA: Leark et al., 2007), or the Conners CPT-II (Conners and Staff, 2000).

Among the best-known individual tests for measuring cognitive flexibility and set-shifting in children is the Children’s Color Trails Test (Llorente, 2003) and the Wisconsin Gard Sorting Tests (Grant and Berg, 1948). For the measure of planning the Tower of Hanoi is widely used (Goel and Grafman, 1995). According to Fernández et al. (2014), the most widely studied executive batteries in children are the Behavioral Assessment of the Dysexecutive Syndrome for Children battery (BADS-C: Emslie et al., 2003) and the Cambridge Neuropsychological Automated battery (CANTAB: Luciana, 2003). Another widely used computerized behavioral battery is the Delis-Kaplan Executive Function System (D-KEFS; Delis et al., 2004). It consists of nine verbal and non-verbal EF tasks that are norm-referenced and appropriate for children and adults (ages 8-89). It is nuanced and tries to explain why a child is performing at a particular level (Diamond, 2020). The NEPSY II (Korkman et al., 2007) is also an interesting and expanding battery in the field of developmental neuropsychology. Based on Luria’s theories, it was designed as a broad neuropsychological measure (Holcomb and Davis, 2011).The NEPSY-II contains 32 different attention and EF subtests designed for children of various ages; many of them are designed for particular age groups.

In addition to these performance-based tests, in recent decades, a new type of assessment based on daily living activities has emerged. These tests, which are also performance-based, involve performing everyday tasks to replicate conditions like those found in real-world settings and aim to prove the ecological validity criticized in conventional cognitive tests for so long now. As per Fernández et al. (2014), Children’s Kitchen Task Assessment is the best-known test for children and adolescents (CKTA: Rocke et al., 2008). According to the authors, the CKTA is a performance-based assessment of EF in children aged six and older. It is a safe, age-appropriate, and goal-directed activity for children, of making playdough. The CKTA measures EF in the following areas: initiation, planning and sequencing, organization, judgment and safety, and completion. The amount and types of cues the child received to complete the activity affect the scoring on the CKTA. In total, there are 15 possible steps for the child to complete and five possible levels of cueing. Each cue is given twice before moving to the next level of cueing. Each level of the cue has a higher point value score. Therefore, higher scores indicate lower levels of executive functioning (Rocke et al., 2008).

Table 1 provides a sample of the most known performance-based tests assessing EF in school-aged children. A comprehensive review of these performance cognitive tests can be found in Goldstein and Naglieri (2014).

**TABLE 1:** Performance-based tests assessing executive functions (EF) in school-aged children.

Test	Age (year)	Time of admin (minutes)	Format	EF components
Digit span tests (Wechsler, 2014) Corsi’s block-tapping test (Corsi, 1972)	6≤	5-10	Individual performance-based test	Working Memory
The dot matrix within the automated working memory assessment battery (Alloway, 2007)	5-11	10-15		
The n-back pictorial paradigm (Isquith et al., 2014)	≥6	5-10		
Stroop test (Stroop, 1992)	5-14	10-15	Individual performance-based test	Inhibition, impulse control, cognitive flexibility
Five digit test (FDT: Lang, 2002)	5-14	10-15		
Day-night Stroop test (Gerstadt et al., 1994)	4-12	5		
Animal Stroop test (Wright et al., 2003)	5-14	10		
Continuous performance test (CPT) and CPT-II (Conners and Staff, 2000)	6≤	14	Individual performance-based test	Sustained attention and inhibition
TOVA (Test of variables of attention; Leark et al., 2007)	4-80	23		
Children’s color trails test (CCTT; Llorente, 2003) Comprehensive trail-making test (CTMT2; Reynolds, 2019) Wisconsin card sorting tests (Grant and Berg, 1948)	8-16 8-80 7≤	10-14 5-15 15-30	Individual performance-based test	Sustained attention, sequencing, cognitive flexibility (or set shifting) Perseveration, abstract reasoning and and problem-solving
Tower of Hanoi (Goel and Grafman, 1995)	5≤	15-20	Individual performance-based test	Planning, cognitive flexibility
Behavioral assessment of the dysexecutive syndrome for children (BADS-C; Emslie et al., 2003)	7-18	35-45 without the rating scale	Test Battery	Inflexibility and perseveration, novel problem solving, planning, impulsivity, and the ability to moderate behavior based on feedback
CANTAB battery (Cambridge neuropsychological automated battery)	4-90	40-45	Neurocognitive Battery	Many processes & neurocognitive functions: memory and attention, psychomotor and motor speed, reasoning and planning abilities
Delis– Kaplan executive Function system (D- KEFS; Delis et al., 2004)	8-89	90	Performance test Battery	Nine performance subtests as a complete battery or individual tests
NEPSY- II (Korkman et al., 2007)	3-16	45– 60	Performance test battery	EF subtests: auditory attention and response, animal sorting, set, clocks, design, fluency, inhibition, and statue

### Rating scales of executive functions

The EF rating scales are an eco-valid indicator of competence in complex, everyday problems (Roth and Gioia, 2005). These rating scales are based on the assumption that they measure behaviors that are associated with processes evaluated by performance-based EF measures.

In their review, Toplak et al. (2013) found that the Behavior Rating Inventory of Executive Function (BRIEF: Gioia et al., 2000) is one of the most commonly used measures of EF. In 2015, these authors published the BRIEF-2 which is a recent version of the BRIEF. The BRIEF-2 includes rating forms for teachers and parents (for ages 5-18), and a self-report form for children and adolescents (for ages 11-18), designed to measure executive functioning in home and school environments. The parent and teacher questionnaires each have 63 items that are rated using a three-point scale (never, sometimes, often) and require 10 minutes to complete for each. The BRIEF-2 is made up of three indices: The Behavioral Regulation Index (BRI), Emotional Regulation Index (ERI) and the Cognitive Regulation Index (CRI). The BRI is comprised of Inhibit and Self-Monitor subdomains. The ERI includes the Shift and Emotional Control, and the CRI comprises the following subdomains: WM, Initiate, Organization of Materials, Task Monitor, and Plan/Organize. Combined, the BRI, ERI, and CRI form the Global Executive Composite (GEC). Results are reported using t-scores relative to the normative sample (*M* = 50; *SD* = 10). T-scores greater than 65 are considered clinically significant. The BRIEF-2 also includes three validity scales, an inconsistency scale (acceptable, questionable, inconsistent), Infrequency and a negativity scale (acceptable, elevated, highly elevated).

Likewise, the Behavior Assessment System for Children, Third Edition (BASC3; Reynolds et al., 2015) is a behavioral assessment instrument that has several features making it one of the most advanced and reliable systems available. It consists of a multimethod, multidimensional set of evaluation tools used for assessing children, adolescents, and young adults between the ages of 2 and 21. The BASC3 components could be used individually or in any combination and could include a Teacher Rating Scale (TRS) and a Parent Rating Scale (PRS), a Self-Report of Personality (SRP), a Structured Developmental History (SDH) and a Student Observation System (SOS) form. The composite scores for the TRS and PRS measure adaptive skills, behavioral symptoms index, externalizing problems, and internalizing problems; the TRS also includes the school problems composite score that measures the effects of school problems. A composite PRS score includes school problems, personal adjustment internalizing problems, inattention/hyperactivity, functional impairment index and emotional symptoms index. Altmann et al. (2017) stated that BASC-3 scales and composites are reliable and have high internal consistency. The alpha coefficients of BASC-3 subscales and composites regularly exceed 0.80, which makes them suitable for diagnostic and treatment purposes.

The Behavioral Assessment of the Dysexecutive Syndrome in Children (BADS-C: Emslie et al., 2003) is also one of the most used questionnaires in studies of EF in school-aged children (Wallisch et al., 2018). The BADS-C is an executive functioning test for children which is an extension of the adult Behavioral Assessment of Dysexecutive Syndrome (BADS) for people aged 16 to 87. The authors aimed to develop a valid and ecologically applicable instrument for predicting executive problems and their severity in daily life. This test assesses all aspects of the Dysexecutive Syndrome: inflexibility, initiative, perseverance, planning, and using feedback to moderate behavior. In addition to assessing a child’s level of competence in each situation, the examiner should also observe how the task was done. Scoring aims to display the qualitative diversity of observational data.

The BADS-C consists of six tests administered individually to children and adolescents with neurodevelopmental disorders such as ADHD, Pervasive Development Disorder, and Traumatic Brain Injury, which are standardized and normed for children and adolescents aged 7-16. Among the six sub-tests are The Playing Card Test, which assesses cognitive flexibility; The Water Test, which assesses problem-solving strategies; The Key Search Test, which evaluates children’s ability to develop a systematic, action-oriented plan, monitor their performance, and consider variables that are not indicated. The Zoo Map Tests 1 and 2 involve two consecutive planning exercises; the first is an open-ended task, and the second measures task scheduling and performance monitoring.

The Dysexecutive Questionnaire for Children (DEX-C) is an 20-item Likert scaled questionnaire that assesses emotional/personality, motivation, behavior, and cognitive difficulties in children and adolescents with dysexecutive syndrome (Roy et al., 2015). Other inventories and questionnaires have also been cited in studies of EF in children, including but not limited to, the Childhood Executive Functioning Inventory (CHEXI: Thorell and Nyberg, 2008) and the Delis Rating of Executive Functions (D-REF: Delis, 2012).

Table 2 summarizes the above-mentioned rating scales assessing EF. A more detailed and exhaustive description of these questionnaires can be found in Goldstein and Naglieri (2014).

**TABLE 2:** Rating scales and inventories assessing executive functions (EF) in school-aged children.

Test	Age (year)	Time of admin (minutes)	Format	Descriptive classification (*T scores*)	EF component
The behavioral rating inventory of executive function (BRIEF-2; Gioia et al., 2015).	5-18	10	Parent or teacher rating scale or adolescent self-report	-T-scores of 60–64 in the mildly elevated range -T scores 65-69 potentially clinically elevate -T scores ≥ 70 clinically elevated	-The Behavioral Regulation Index (BRI): Inhibit & Self-Monitor -The Emotional Regulation Index (ERI): Shift & Emotional Control -The Cognitive Regulation Index (CRI): WM, Initiate, Organization of Materials, Task Monitor, & Plan/Organize
Dysexecutive questionnaire for children (DEX-C) from (BADS-C; Emslie et al., 2003)	7-16	15	20-item Likert-scaled Caregiver/teacher completed questionnaire	-Higher scores indicate more problems, cut-off scores not specified	Emotional/personality, motivational, behavioral, and cognitive difficulties associated with Dysexecutive Syndrome
Childhood executive functioning inventory (CHEXI; Thorell and Nyberg, 2008)	4-12	5	Rating instrument	-No normative data is available & has not yet been standardized	WM, planning, regulation, and inhibition
The behavior assessment system for children –third edition (BASC-3; Reynolds et al., 2015)	2-21 11 months	10-20	Parent, teacher, or self-Report	-T-scores (M = 50, SD = 10)	Behaviors and emotions of children and adolescents: adaptive skills, behavioral symptoms index, externalizing problems, and internalizing problems
Delis rating of executive functions (D-REF; Delis, 2012)	5-18	5-10	Parent, teacher, or child reports	-T scores 10–54 within the normal range -T-scores of 55–59 borderline elevated -T-scores of 60–70 mildly to moderately elevated range -T scores ≥ 70 severely elevated & indicate significant symptoms	Three index scores: behavioral, emotional, and cognitive functioning + total composite score

### Performance-based tests vs. rating scales: Pros and cons

These tests measure specific EF under controlled conditions using computerized and/or paper-pencil tasks (Muris et al., 2008). Psychometric research has demonstrated that tests from the cognitive sciences can provide significant and unique insights into latent estimates of global and specific executive functioning abilities (Miyake et al., 2000; Willoughby et al., 2016). Their concurrent and predictive validity, sensitivity and specificity has been supported by numerous cognitive, developmental, and clinical studies that have demonstrated strong convergence with functional outcomes that are ecologically valid (Miyake et al., 2000; Wells et al., 2018). This concurrent and predictive validity evidence includes experimental and longitudinal linkages between EF performance tests, objective and subjective measures of inattention, hyperactivity, impulsivity, emotion recognition, reading skills, math performance, social skills, organizational skills, parent-child relationship quality, and activities of daily living (Karalunas et al., 2017; Wells et al., 2018).

Harvey (2012) asserted that performance-based tests are the core of neuropsychological assessment. This method can be used for many purposes, such as collecting diagnostic information, identifying differential diagnoses, assessing treatment response, and predicting functional recovery and potential self-reports of functioning, along with observations of behavior during testing, are crucial aspects of testing data.

According to Soto et al. (2020), compared to rating scales, performance-based tests have been criticized as having poor generalizability and ecological validity since scores may reflect optimal performance under controlled conditions that are not representative of real-world situations in which EF guide behavior (e.g., poor external/face validity). Performance-based EF tests may also take longer to administer than rating scales (Toplak et al., 2013; Toplak et al., 2017).

Regarding the EF rating scales, Muris et al. (2008) and Toplak et al. (2013) assert that they are less time-consuming measures, which are completed by knowledgeable observers (e.g., parents, teachers) that are assumed to capture EF as they are implemented in everyday, real-world settings. Rating scales can collect data from experienced informers in a variety of contexts and circumstances. The collected data can be used to identify behaviors that occur very infrequently in ways that respect the privacy of the individual. On the other hand, direct observation or testing might impinge on the individual’s daily life (Barkley and Murphy, 2011).

Rating scales have many advantages, showing supported convergent validity (McAuley et al., 2010; Gross et al., 2015). It has also been demonstrated that EF rating scales have concurrent and predictive validity. In fact, they predict theoretically related ratings of personality traits, social skills, internalizing and externalizing disorders, mood difficulties and academic performance (Gross et al., 2015; Buchanan, 2016; Gerst et al., 2017). There is, however, criticism of the construct validity of EF rating scales since their correlation with performance-based tests that are supposedly measuring the same construct is inconsistent or weak (Gross et al., 2015; Nordvall et al., 2017; Soto et al., 2020). Furthermore, the content validity of these scales has been questioned. Toplak et al. (2013) concluded that EF rating scales measure the success of goal pursuit or externalizing behaviors rather than cognitive functioning as intended. Barkley and Murphy (2011) highlighted other rating scales’ inherent disadvantages. The first assumption is that both the respondent and the examiner understand the nature of the item being assessed. Then, testing hypotheses that require considerably more accuracy than the scale can provide may be hampered by the generality or ambiguity of the item being evaluated and finally, confounding factors (e.g., IQ, education, emotional status, life experiences, previous experience with similar rating scales) may affect the rater’s ability to accurately report the behavior indicated on the scale.

In summary, both the EF Performance-Based Tests and Rating Scales have their inherent advantages and disadvantages. Each provides different but complementary information necessary in the EF assessment. According to Toplak et al. (2013) and Toplak et al. (2017), the performance-based tests provide valuable insight into cognitive processes and their efficiency in structured environments. However, EF ratings are helpful in predicting occupational performance and academic performance because they provide information about goal-directed behavior in everyday situations.

### Challenges to executive functions assessment in school-aged children

Several challenges have been outlined in the assessment of EF, especially in children. Below is a brief overview of the major challenges that should be considered when assessing and interpreting the assessment outcomes.

### Impurity of executive functions measures

A central problem in the assessment of EF, measurement errors and the multifactorial nature of executive tasks is the parallel involvement of “lower-level” processes. This problem also extends to the involvement of the several executive processes themselves. Indeed, executive tasks simultaneously involve several executive and non-executive processes whose interaction is probably differing according to the measures and methodological parameters chosen. This multi-composite nature of executive tests is inevitably amplified in children due to developmental dynamics that accentuate the mutual interaction of these two levels of processes. The problem with many EF tests is they are commonly affected or contaminated by general cognitive ability or intelligence level (Barkley and Murphy, 2011; Toplak et al., 2013). Therefore, to reduce task impurity and low reliability, Friedman and Miyake (2017) recommend that the latent factor approach and variables such as socioeconomic status (SES) and IQ should be statistically controlled and considered as covariates.

### Contextual and cultural factors

Many studies suggested contextual and cultural influences on the early development of cognitive control skills in children urging that these factors needed to be considered when assessing EF (Schmitt et al., 2018; Obradović and Willoughby, 2019; Schirmbeck et al., 2020; Roukoz et al., 2021). One of the most used constructs, evaluating the impact of environmental context, is socioeconomic status (SES). It is a bundle of social and economic factors affecting education, health, and psychological well-being (Guerra et al., 2022). Predictors of SES include family income, type of school (public or private), parental education and/or occupation, or a combination of these factors. A child’s neuropsychological development could be affected by these factors (Guerra et al., 2020). Higher SES has been associated with better executive performance, while lower SES has been associated with poorer executive performance (Obradović and Willoughby, 2019; Schirmbeck et al., 2020; Guerra et al., 2020; Guerra et al., 2022). Blair and Raver (2015) hypothesized that these factors are likely mitigated by more complex underlying mechanisms, like prenatal factors, nutrition, and stress. The authors postulated that early adversity and poverty-related risks cause marked changes to a child’s stress response system, which in turn affects the neural systems essential to EF development.

Moreover, EF development is likely to be significantly influenced by the environment in which children grow up (Guerra et al., 2020). According to Schmitt et al. (2018), differences across countries have been attributed to (i) pedagogic approaches and educational settings (as teacher’s beliefs about play and learning or classroom management), (ii) parental upbringing and expectations (as maternal sensitivity, scaffolding, and autonomy support) (iii) and social norms and value systems (as individualist versus collectivist society). It has been shown that bilingual children outperform monolingual children across countries (Schirmbeck et al., 2020). According to other studies, East Asians outperformed Western counterparts on direct assessment measures of EF but not on parent and teacher ratings of children’s EF from preschool through adolescence (Schirmbeck et al., 2020). Compared to parents in other countries, Chinese parents rated their children’s EF lower in the latter study. The possibility is that high parental expectations of their children’s cognitive and behavioral self-regulation might result in parents strengthening these skills, which in turn might lead to improved children’s EF abilities.

### Validity issues

Most executive tests have been developed for adults and applied to children by assuming that they measure comparable executive processes and that they can detect similar neuroanatomo-functional dysfunctions (Chan et al., 2008). However, immature performance may be wrongly judged to be deficient, especially if pathology is suspected, when it may rather reflect a normal developmental trajectory. Another significant problem is the lack of standardized measures of EF for young children with appropriate normative data that can lead to biased interpretation (Young et al., 2017).

In low- and middle-income countries, validity issues are more prominent. The majority of direct assessments of EF in young children were developed in high-income countries. Thus, EF tasks need to be adapted to the culture and context of the country (Obradović and Willoughby, 2019). A rigorous adaptation process needs to be conducted, based on normative data specific to the target population, in order to achieve psychometric validity and reliability (Bellaj et al., 2018). Ideally, it should involve community experts proficient in local languages and cultures, as well as experience with the target population. Assessing EF ecologically requires the involvement of local experts (Obradović and Willoughby, 2019).

### A questionable ecological validity of the executive functions performance-based measures

Executive functions tests also pose a challenge as the best way to assess EF since they have very low or no ecological validity. It is therefore unlikely that they correlate well with daily life activity ratings in adults or children with frontal lobe lesions, TBI, or other neurological anomalies (Gioia et al., 2000; Gioia et al., 2002; Isquith et al., 2014). Therefore, the validity of EF rating scales and performance-based tests appears to be divergent rather than convergent. Toplak et al. (2013) in their meta-analysis of research on this issue demonstrated that most EF tests measure different constructs than an EF rating or a direct evaluation of EF in daily life. It has even led some researchers to consider EF tests as not accurately assessing EF, daily life activities, or impairment in major domains of life because of this significant failure to correlate well with EF ratings (Barkley and Murphy, 2011).

In his conception of EF as an extended phenotype, Barkley (2012) claims that performance-based measures of EF reflect self-directed, internalized pre-executive processes that promote self-control at the instrumental-cognitive level of EF. However, EF’s rating scales or ecological measures reflect functioning at a strategic-cooperative level which involves achieving long-term goals in areas such as education, work, cohabitation, childrearing, financial management, driving, and community. Goals span longer periods, require broader social collaborations, and involve more complex behaviors and interactions. This explains their weak relationship with performance-based tests. This conception of EF levels appears congruent with the proposition that different EF assessment methods capture different aspects of behavioral and cognitive functioning (Toplak et al., 2013; Toplak et al., 2017).

## Limitations

This review has some limitations and it is far from being comprehensive, both in terms of the models and theories of this construct, as well as the development studies across the life span, and the wide variety of measures, particularly those related to the hot component, that have not been fully examined. We were able to sort out a maximum number of articles by using “executive functions” as keywords, but some relevant papers may have been missed. Our review is also not systematic and does not meet pre-specified eligibility and selection criteria. Indeed, our topic required an in-depth narrative review along with adhering to quality criteria by ensuring that it is (i) clear and synthetic with easy-to-interpret tables and figures (ii) rigorous with minimal selection bias, including relevant publications without being limited to the experimental studies privileged in systematic reviews (iii) and finally integrative and synthesizing the knowledge and gaps identified.

## Conclusion

Executive functions are predictors of children’s and teenagers’ behavior and performance in a variety of contexts, including education, family, and social relationships. This construct has been frequently marred by inconsistency, ambiguity in conceptualization and challenges related to the assessment. However, we similarly found commonalities in the definition and theorization of this umbrella term, where most definitions emphasize the goal-directed nature of EF and the wide variety of processes involved essentially WM, inhibition, cognitive flexibility, and planning.

The assessment of EF remains a hotly debated topic due to all the issues and challenges discussed in the previous sections on reliability and ecological validity. These problems of EF measurement tools are amplified in children given the impact of maturity as well as all the neuroanatomical, biological, environmental, and cultural factors that could influence the development of these functions. Thus, a more nuanced clinical analysis of EF requires a variation in methodological parameters, to promote a “subtraction” of processes and clarify the origin of the multifactorial deficit in an executive task. Moreover, ecological, and ethological approaches adapted to the child’s reality are to be encouraged, whether it is through tests theoretically aligned with everyday function “Verisimilitude” and predicting some aspect of the child’s everyday life “Veridicality”, or by measuring the child’s real behavior in the context of his/her life through questionnaires or quantitative and qualitative observations.

Executive functions may refer to a set of diverse and interdependent functions ensuring the control and regulation of cognitive and socio-affective goal-directed activities. In addition to acting on different processes in a global and age-specific way, they are sensitive to biological, environmental, and sociocultural influences. As EF conceptualization and operationalization are complex there cannot be a single “silver bullet” test or definition. Hence, researchers should use precise and consistent language when defining the construct and align references with definitions as well. Clinicians should strive to ensure comprehensive assessment based on multiple informants and standardized, reliable, ecologically valid, context-sensitive, and appropriate measures. To facilitate the development and operationalization of EF processes in school-aged children, future research is needed to define, understand, and design appropriate measures, clinical strategies, and interventions.

## Author contributions

SS wrote the first draft of the manuscript. KC and TB wrote sections of the manuscript. All authors contributed to manuscript revision, read, and approved the submitted version.
